# Iron-rich food consumption and predictors among children aged 6–59 months old in Ethiopia: A multilevel complex sample analysis of the Ethiopian mini-demographic and health survey 2019 data

**DOI:** 10.1371/journal.pone.0305046

**Published:** 2024-06-04

**Authors:** Girma Beressa, Fikreab Desta, Bikila Lencha, Biniyam Sahiledengle, Daniel Atlaw, Degefa Gomora, Demisu Zenbaba, Eshetu Nigussie, Neway Ejigu, Tamiru Yazew, Telila Mesfin, Kenenisa Beressa

**Affiliations:** 1 Department of Public Health, Madda Walabu University, Goba, Ethiopia; 2 School of Medicine, Madda Walabu University, Goba, Ethiopia; 3 Department of Midwifery, Madda Walabu University, Goba, Ethiopia; 4 Department of Public Health, Salale University, Fitche, Ethiopia; 5 Department of English, Addis Ababa University, Addis Ababa, Ethiopia; MeU: Mattu University, ETHIOPIA

## Abstract

**Background:**

Children with inadequate iron consumption had slower growth, weaker immunity, and poor cognitive development. Although the public health importance of iron-rich consumption in Ethiopia is known, evidence for iron-rich food consumption and predictors among children aged 6–59 months old in Ethiopia is sparse. This study aimed to assess iron-rich food consumption and predictors among children aged 6–59 months old in Ethiopia.

**Methods:**

This study used Ethiopia mini demographic and health survey 2019 (EMDHS-2019) data with a total weighted sample size of 5,112 among children aged 6–59 months old. A multilevel mixed effect logistic regression analysis was used to identify predictors of good iron-rich food consumption.

**Results:**

The proportion of good consumption of iron-rich foods among children aged 6–59 months was 27.99% (24.22, 32.10%). The findings revealed that children born to mothers who completed primary education [AOR = 1.88, 95% CI: 1.11, 3.19], a higher education [AOR = 4.45, 95% CI: 1.28, 15.48], being born to the poorer family [AOR = 1.89, 95% CI: 1.04, 3.43], richer [AOR = 2.12, 95% CI: 1.03, 4.36], and richest [AOR = 3.57, 95% CI: 1.29, 9.93] were positively associated with good iron-rich food consumption among children aged 6–59 months old. Nevertheless, being 24–59 month-old children [AOR = 0.58, 95% CI: 0.44, 0.72], residents of the Afar [AOR = 0.23, 95% CI: 0.08, 0.67], Amhara region [AOR = 0.30, 95% CI: 0.14, 0.65], and Somali region [AOR = 0.01, 95% CI: 0.01, 0.07] were negatively associated with good iron-rich food consumption among children aged 6–59 months old.

**Conclusion:**

The finding revealed that there was low consumption of iron-rich foods among children aged 6–59 months in Ethiopia compared to reports from East African countries. Improving women’s literacy and economic empowerment would improve iron-rich food consumption among children aged 6–59 months old. This study’s findings would have implications for policymakers in Ethiopia to enhance iron-rich food consumption.

## Introduction

The World Health Organization (WHO) reported a 42.6% global incidence of anemia among children, making it the most frequent micronutrient deficit, affecting over 2 billion people [1]. Ethiopia’s Demographic and Health Survey (EDHS) found that 44% of children aged 6–59 months are anemic, with iron deficiency accounting for half of these cases. Iron deficiency is mostly caused by poor consumption, limited absorption, and infections [2]. Anemia in children under the age of five has several consequences, including decreased mental function, limited tolerance to infection, and mortality from anemic heart failure [3].

Previous study reveal that preschool children with inadequate iron consumption had slower growth, weaker immunity, and worse cognitive development [4]. Other existing studies indicate that the proportion of iron-rich food consumption in early children ranges from 21.41% in low- and middle income countries (LMICs) to 90% in high-income countries (HICs) [5,6]. Individual-level parameters such as low antenatal care (ANC) visits, institution delivery, child age, gender, and birth order, as well as community-level variables such as regions and community women’s education, all demonstrated a high link with iron consumption among Ethiopian children aged 6–59 months [5,7].

Global attempts to prevent anemia focus on boosting iron consumption, as iron deficiency plays a significant role [8]. The World Health Organization (WHO) recommends daily iron consumption as a recommended method for the treatment and prevention of iron deficiency anemia (IDA) [9,10]. Although animal-source foods (ASF) are high in protein, fat, and minerals, their high cost limits their use in Ethiopia. For this reason, low-income countries, including Ethiopia, lack the physical access and economic capacity to acquire fortified animal products. Micronutrient deficits, such as anemia, can develop from a lack of animal-based meals [11]. Identifying significant individual and community-level predictors of iron-rich food intake is crucial to improving iron-rich consumption in Ethiopia. This study’s findings would have implications for policymakers in Ethiopia to improve iron-rich food intake. Thus, this study aimed to assess the proportion of iron-rich food consumption and individual and community-level predictors among 6–59-month-old children in Ethiopia using Ethiopia Mini Demographic and Health Survey (EMDHS-2019) data.

## Methods

### Study design, data source, and subjects

The data used for this study were retrieved from the Ethiopian Demographic and Health Survey (EDHS, 2019), which was employed using a community-based cross-sectional study. The source population consisted of all mother-child pairs aged 6–59 months, whereas the study population consisted of all chosen or sampled living children aged 6–59 months who lived with their mother. Women aged 15–49 with children ages 6–59 who were permanent residents or visitors who stayed in the houses the night before the survey were eligible for interviews.

The standard EDHS data set has a large sample size, which helps to obtain parameters [12]. The mini EDHS used a two-stage sampling approach to collect data from nine Regional States and two City Administrations. In the first step, 305 enumeration areas (EAs) (93 in urban areas and 212 in rural areas) were chosen with a probability proportionate to their size based on the 2019 Ethiopia population and housing census (EPHC) frame and independent selection in each sample strata. In the second stage of selection, a predefined number of 30 houses per cluster were chosen with an equal chance of systematic selection from the newly produced household list. followed by interviews with chosen mother-child pairs. The Ethiopian Demographic and Health Survey results, published on the Measure DHS website, include a thorough sampling technique (www.dhsprogram.com). The dataset is available at https://dhsprogram.com.

## Data collection instruments

Child age, child sex, marital status, religion, maternal education, wealth index, and birth order were individual-level factors, whereas residence and region were community-level factors [2]. The wealth index was determined using the principal components analysis based on the number and types of consumer goods they own, ranging from a television to a bicycle or car, as well as housing characteristics such as source of drinking water, toilet facilities, and flooring materials [2,13].

### Outcome assessment

If the children aged 6–59 months living with their mother consumed at least one iron-rich food at any time in the 24 hours preceding the interview, among four food items, eggs, organ meat (liver, heart, or other organs), meat (beef, pork, lamb, or chicken), and fish were considered good consumption, otherwise poor consumption [12].

### Data processing, model building, and analysis

Data analyses were conducted using Stata^TM^ version 14 [14]. Descriptive statistics such as frequency and percentages were used to describe study subjects. The proportion and frequencies were weighted. All analyses employed the individual sample weight (v005/1,000,000) to adjust for over- and under-sampling. The EDHS dataset is hierarchical, with children nested in households and households within clusters. Multicollinearity was checked among predictors using a correlation matrix (R). Variables in bivariable multilevel mixed effect logistic regression analyses less than 0.25 were entered into multivariable multilevel mixed effect logistic regression analyses to control potential confounding effects. Four models were fitted: the null model (model without predictors); model 1: individual-level factors; model 2: community-level factors; and model 3: individual and community-level factors. Multilevel mixed effects logistic regression analyses were used to examine the association between individual and community-level factors and iron-rich food consumption (yes = 1, no = 0). A complex sample survey multilevel mixed effects logistic regression data analysis technique (melogit [pweight = swt] || v001:) was used to analyze the data. The Stata command "svy" was used to establish survey data and estimate the percentage of iron-rich food consumption. To quantify the strength of the association between predictors and iron-rich food consumption, an adjusted odds ratio (AOR) along with a 95% confidence interval (CI) was used. The final model was evaluated for goodness-of-fit using the Akaike Information Criterion (AIC), Bayesian Information Criterion (BIC), and log-likelihood ratio (LLR). The model with the lowest AIC and BIC and highest LLR was considered the best fit.

The Median Odds Ratio (MOR), defined as the median value of the odds ratio between the areas at the lowest and highest risk when two clusters are randomly chosen, was used to quantify variation. MOR = e0.95√VA or exp. [√ (2 × VA) × 0.6745], where VA represents the area-level variation. The proportional change in variance (PCV) measures the variance in iron-rich intake among children aged 6–59 months, which is explained by several variables. The PCV is computed as Vnull-VA/Vnull* 100. Where Vnull is the initial model’s variance and VA is the model’s variance with added terms. The intraclass correlation coefficient (ICC) measures the variation in iron-rich intake between clusters. It is calculated as ICC = VA ÷ VA + 3.29 * 100%, where VA = area/cluster level variance [15]. A p value less than 0.05 was declared statistical significant.

### Ethical approval

Ethical approval was obtained from the Ethiopian Health and Nutrition Research Institute Review Board. Informed verbal permission was obtained from each woman. Ethical permission was received from Measure DHS using a data access request form. The EDHS data is available to the general public in various formats upon request from the Measure DHS website (www.measuredhs.com). All approaches followed the relevant tenets of the Helsinki Declaration.

## Results

### Socio demographic and economic factors

A total weighted sample of 5,112 mother-child pairs was included in this study from the EDHS dataset. Three-fourths, 3,916 (76.60%) of study subjects were rural residents. Nearly two-thirds of 3,513 (68.72%) of children were 24–59 months old. Half, 2,638 (51.60%), of the study subjects were Muslims. Most 2,834 (55.44%) of children’s mothers had no education. Most 1,119 (33.90%) of the study subjects were in the in the poorest wealth quintile (Table 1).

**Table 1:** Socio demographic and economic factors of study subjects, Ethiopia, 2019 (N = 5,112).

Variables	Weighted frequency (n)	Weighted percent (%)
**Child age**		
6–23	1,599	31.28
24–59	3,513	68.72
**Sex**		
Male	2,656	51.96
Female	2,456	48.04
**Marital status**		
Never in union	21	0.41
Married	4,755	93.02
Live with partner	35	0.68
Widowed	57	1.12
Divorced	167	3.27
Separated	77	1.57
**Religion**		
Orthodox	1,444	28.25
Catholic	29	0.57
Protestant	944	18.47
Muslim	2,638	51.60
Traditional	57	1.12
**Maternal education**		
No education	2,834	55.44
Primary education	1605	31.40
Secondary education	409	8.00
tertiary education	264	5.16
**Wealth quintile**		
Poorest	1,733	33.90
Poorer	884	17.29
Middle	722	14.12
Richer	654	12.79
Richest	1,119	21.89
**Birth order**		
1	1,111	21.73
2–3	1,698	33.22
≥ 4	2,303	45.05
**Residence**		
Urban	1,196	23.40
Rural	3,916	76.60
**Region**		
Tigray	395	7.73
Afar	574	11.23
Amhara	465	9.10
Oromia	637	12.46
Somali	569	11.13
Benishangul Gumuz	475	9.29
SNNPR	586	11.46
Gambela	385	7.53
Harari	402	7.86
Addis Ababa	265	5.18
Dire Dawa	359	7.02

SNNPR: Southern Nations, Nationalities, and Peoples’ Region.

### Proportion of iron-rich food consumption

The proportion of iron-rich food consumed among children aged 6–59 months in Ethiopia was 27.99% (24.22, 32.10%).

### Individual and community-level predictors of iron-rich food consumption among children aged 6–59 months old

The ICC for the null model was 38.52%. This revealed that there was heterogeneity in the iron-rich foods intake of children aged 6–59 months among clusters. The full model had the lowest AIC and highest LLR, hence it was chosen as the best fit model. The multivariable multilevel mixed effect logistic regression analyses revealed that being 24–59 month-old children [AOR = 0.58, 95% CI: 0.44, 0.72], children born to mothers Catholic followers [AOR = 0.01, 95% CI: 0.01, 0.08], children born to mothers completed primary education [AOR = 1.88, 95% CI: 1.11, 3.19], a higher education [AOR = 4.45, 95% CI: 1.28, 15.48], being born to the poorer family [AOR = 1.89, 95% CI: 1.04, 3.43], richer [AOR = 2.12, 95% CI: 1.03, 4.36], richest [AOR = 3.57, 95% CI: 1.29, 9.93], and residents of the Afar [AOR = 0.23, 95% CI: 0.08, 0.67], Amhara [AOR = 0.30, 95% CI: 0.14, 0.65], and Somali region [AOR = 0.01, 95% CI: 0.01, 0.07] were significantly associated with good iron-rich food consumption among children aged 6–59 months old (Table 2).

**Table 2:** Multivariable multilevel mixed effect logistic regression analyses of iron-rich food consumption and predictors among children aged 6–59 months old, Ethiopia, 2019 (N = 5,112).

Variable	Null model^a^	Model 1^b^	Model 2^c^	Model 3^d^
	AOR (95% CI)	AOR (95% CI)	AOR (95% CI)	AOR (95% CI)
C-age (months)				
6–23		Ref		Ref
24–59		0.58 (0.45, 0.73		**0.57 (0.44, 0.72)** **
Sex				
Male		Ref		Ref
Female		1.37 (0.94, 2.01)		1.37 (0.94, 2.01)
**Marital status**				
Never in union		Ref		Ref
Married		0.27 (0.02, 3.57)		0.28 (0.02, 3.68)
Live with partner		0.29 (0.01, 7.07)		0.32 (0.01, 8.40)
Widowed		1.42(0.06, 35.49)		1.50 (0.05, 41.33)
Divorced		0.11 (0.01, 2.75)		0.12 (0.01, 2.97)
Separated		0.71(0.01, 26.91)		0.63 (0.01, 22.12)
**Religion**				
Orthodox		Ref		Ref
Catholic		0.01 (0.01, 0.10)		**0.01 (0.01, 0.08)** **
Protestant		1.29 (0.50, 3.35)		1.06 (0.34, 3.32)
Muslim		0.94 (0.44, 2.04)		1.32 (0.54, 3.21)
Traditional		0.19 (0.01, 2.52)		0.16 (0.01, 2.05)
**M-edu**				
No education		Ref		Ref
Primary edu		1.98 (1.17, 3.36)		**1.88 (1.11, 3.19)** *
Secondary edu		1.17 (0.52, 2.63)		1.13 (0.50, 2.53)
higher edu		4.31 (1.25, 2.52)		**4.45(1.28,15.48)** *
**Wealth quintile**				
Poorest		Ref		Ref
Poorer		2.36 (1.17, 3.36)		**1.89 (1.04, 3.43)** *
Middle		1.51 (0.68, 3.35)		1.27 (0.58, 2.80)
Richer		2.55 (1.24, 5.24)		**2.12 (1.03, 4.36)** *
Richest		4.01 (1.67, 9.58)		**3.57 (1.29, 9.93)** *
**Birth order**				
1		Ref		Ref
2–3		0.91 (0.60, 1.41)		0.91 (0.60, 1.41)
≥ 4		0.77 (0.45, 1.31)		0.77 (0.44, 1.31)
**Residence**				
Urban			Ref	Ref
Rural			0.51 (0.30, 0.87)	1.34 (0.59, 3.04)
**Region**				
Tigray			Ref	Ref
Afar			0.18 (0.09, 0.36)	**0.24 (0.08, 0.67)** *
Amhara			0.32 (0.16, 0.62)	**0.30 (0.14, 0.65)** *
Oromia			0.82 (0.45, 1.48)	0.74 (0.28, 1.95)
Somali			0.01 (0.01, 0.04)	**0.01 (0.01, 0.07)** *
Benishangul			0.57 (0.31, 1.08)	0.66 (0.28, 1.58)
SNNPR			1.10 (0.60, 2.03)	1.01 (0.35, 2.94)
Gambela			0.34 (0.16, 0.72)	0.32 (0.09, 1.18)
Harari			0.60 (0.31, 1.11)	0.39 (0.14, 1.08)
Addis Ababa			1.02 (0.47, 2.20)	0.61 (0.23, 1.61)
Dire Dawa			0.63 (0.33, 1.18)	0.46 (0.16, 1.30)
Random effects				
Variance (SE)	1.83 (0.37)	1.53 (0.34)	1.12 (0.25)	1.15 (0.28)
ICC%	0.39	0.27	0.23	0.21
PCV%	0	16.39%	38.80%	37.15%
MOR	3.60	3.23	1.01	1.01
Model fit statistics				
AIC	2790.10	2621.40	2712.47	2580.35
BIC	2801.95	2751.69	2789.46	2775.35
Log likelihood	-1401.36	-1300.98	-1346.78	-1264.16

* Statistically significant p value < 0.05

** p value < 0.001; Ref: Reference; AOR: Adjusted odds ratio; CI: Confidence interval; SE: Standard error; ICC: Intraclass correlation, PCV: Proportional change in variance; MOR: Median odds ratio; ^a^model without predictors; ^b^adjusted for individual-level factors; ^c^adjusted for community-level factors; ^d^full model; C-age: Child age; edu: Education; M-edu: Maternal education.

## Discussion

This study aimed to assess the proportion of iron-rich food consumption and individual and community-level predictors among 6–59-month-old children in Ethiopia. The findings clearly showed that the proportion of good consumption of iron-rich foods among children aged 6–59 months was 27.99%. Being 24–59 month-old children, children born to mothers who were Catholic followers, children born to mothers who completed primary education or a higher education, being born to the poorer family, richer, richest, and residents of the Afar, Amhara, and Somali region were significantly associated with iron-rich food consumption among children aged 6–59 months old.

The proportion of good consumption of iron-rich foods among children aged 6–59 months was 27.99%. This finding was comparable to a study conducted in Ethiopia [16]. Nevertheless, this finding was higher than studies conducted in Ethiopia [5,7], Rwanda [17], and Afghanistan [18]. On the other hand, this finding was lower than studies carried out in sub-Saharan Africa (SSA) (42.1%) [19], Kenya (33%) [20], Uganda (40%) [21], and Sierra Leone (53.38%) [22]. This might be due to differences in media outlet exposure, socio-cultural and economic status, and study periods.

Children aged 24–59 months old were 43% less likely to consume good iron-rich foods compared to their counterparts. This finding agreed with a study conducted in Ethiopia [5]. A study carried out in Ethiopia also found that as a youngster grows older, the likelihood of having anemic reduces [23]. However, this finding disagreed with a study carried out in India [24]. This might be due to the fact that there are differences in socio-cultural and economic status.

Children born to mothers who completed a higher education level were 4.45 times more likely to consume good iron-rich foods compared to children born to mothers who did not have an education level. This finding agreed with studies carried out in Ethiopia [5,16], Rwanda [17], Afghanistan [18], and India [24]. This could be due to the fact that educated women are exposed to various media outlets and understand the benefits of nutrition for health. Moreover, educated mothers improve health-seeking behavior and receive counselling services. Other comparable research suggests that children born to illiterate mothers are more likely to develop anemia and consume fewer iron-rich foods than children born to educated mothers, and vice versa [5,17,25].

Children born to mothers who had the richest wealth quintile were 3.57 times more likely to consume good iron-rich foods compared to the children born to the poorest mothers. This agreed with studies conducted in Ethiopia [5], Rwanda [17], and SSA [19]. The possible explanation could be that children born to mothers from wealthy families have access to and can afford sources of iron-rich food.

Children born to mothers who resided in the Afar (77%), Amhara (70%), and Somali (99%) regional states of Ethiopia were less likely to consume good iron-rich foods compared to children born to mothers who resided in the Tigray region, Ethiopia. This might be due to differences in the economies of these regions of the country. Because the study was conducted in various places, such as "geographically, culturally, and traditionally," these variations may have an impact on iron-rich food consumption.

### Strength and limitations of the study

Because this dataset is a weighted sample nationwide, it might be indicative of the country. Nevertheless, the data was gathered through self-reports, which might contribute to recall and social desirability bias. Birthplace of the child, maternal age, maternal health conditions, ANC attendance scenario, household empowerment of the mother, status of maternal information, awareness, and health education were not examined. Moreover, since the study is cross-sectional, a cause-and-effect relationship might not be established.

## Conclusion

The finding revealed that there was a low consumption of iron-rich foods among children aged 6–59 months in Ethiopia. Improving women’s literacy and economic empowerment would improve iron-rich food consumption among children aged 6–59 months old. This study’s findings would have implications for policymakers in Ethiopia to promote iron-rich food consumption. Ethiopia should adopt strategies to enhance iron-rich food consumption during these key stages of growth and development.
